# Interstitial 14q24.3 to q31.3 deletion in a 6-year-old boy with a non-specific dysmorphic phenotype

**DOI:** 10.1186/s13039-014-0077-4

**Published:** 2014-11-19

**Authors:** Mariluce Riegel, Lilia MA Moreira, Layla D Espirito Santo, Maria Betânia P Toralles, Albert Schinzel

**Affiliations:** Medical Genetics Service, Hospital de Clínicas de Porto Alegre, Rua Ramiro Barcelos 2350, 90035-003 Porto Alegre, RS Brazil; Postgraduate Program in Genetics and Molecular Biology, UFRGS, Porto Alegre, RS Brazil; Institute of Medical Genetics, University of Zurich, Zurich, Switzerland; Laboratory of Human Genetics and Mutagenesis; Genetics & Society Program, Institute of Biology, Federal University of Bahia (UFBA), Salvador, Bahia Brazil; Postgraduate Studies Program in Interactive Processes of Organs and Systems, Institute of Health Sciences, Federal University of Bahia (UFBA), Salvador, Bahia Brazil

**Keywords:** Genotype-phenotype correlation, 14q interstitial deletion, Array-CGH

## Abstract

**Background:**

Few patients with interstitial deletions in the distal long arm of chromosome 14 have been reported, and these patients showed rather indistinct features, including growth and mental retardation and phenotypic alterations.

**Results:**

We describe a de novo 14q interstitial deletion in a 6-year-old boy with dysmorphic facial traits such as hypertelorism, short and narrow palpebral fissures, broad nose with anteverted nostrils, long philtrum, thin upper lip with cupid’s bow, prominent and everted lower lip, mildly low-set ears, as well as moderate developmental delay and mild mental retardation. Array-CGH mapped the deletion to the region 14q24.3 to 14q31.3, including 13.11 Mb, proximal to the imprinted genomic region of 14q32.

**Conclusion:**

This mild phenotypic presentation suggests that the deleted segment does not contain essential genes for early organ development. Twenty-two genes with known functions, including Neurexin III (*NRXN3*, OMIM 600567), map to the region deleted in the propositus.

## Background

The few reported patients with interstitial microdeletions in the long arm of chromosome 14 have shown a broad and inconsistent spectrum of clinical features caused by loss of different segments of 14q. An understanding of the phenotypes of these clinical disorders is further hampered by the scarcity of reports and the fact that most of the reported patients were diagnosed using only traditional chromosome banding with inaccurate and approximate mapping of the deleted segment.

Cingöz et al. [1], considering the minimal size of these deletions in 11 patients, constructed a map for the three shortest regions of deletion overlap. The proximal region includes the gene encoding Neurexin III (*NRXN3*, MIM 600567), for which haploinsufficiency is associated with cognitive and neurological defects.

Here, we report a male patient with a de novo deletion of the segment 14q24.3 to q31.3, determined using comparative genomic hybridization with microarray (array CGH; aCGH). We also sought to correlate the clinical findings to those of patients with deletions overlapping that identified in the propositus. Considering that breakpoint determination using only banded karyotypes is not precise and may be inaccurate, we included only reports with molecular determination or confirmation of the breakpoints in our comparison.

## Case presentation

The patient was a male aged 6 years and 9 months, the only son of a 38-year-old mother and a 41-year-old father who measured 1.58 m and 1.76 m in height, respectively, and were healthy and nonconsanguineous. The parents reported no genetic conditions or birth defects in the family. The pregnancy was planned and uneventful. Delivery was by cesarean section at 39 weeks and 6 days. The patient’s birth weight was 3145 g, with a length of 49 cm and head circumference of 33.5 cm, which are all appropriate for the gestational age. There was no visible perinatal asphyxia. The postnatal course was characterized by psychomotor delay and muscular hypotonia.

At the first clinical evaluation at 20 months of age, he showed mild dysmorphic features. The first tooth erupted at 8 months of age. Beginning at 6 months of age, he received regular neurological and pediatric follow-ups. The first smile was noted at the age of 2 months, rolling from a ventral to a dorsal position was observed at 3 months, following objects with his eyes was observed at 6 months, maintaining a sitting position was observed at 8 months, maintaining a standing position was observed at 10 months, responding to his name was observed at 10 months, walking was observed at 20 months, and toilet training was achieved at 2 ½ years.

At 6 months of age, physical therapy and an interdisciplinary program of early stimulation were initiated. He was discharged from physical therapy at the end of the second year of life. Psychologic evaluation at 2 years of age showed cognitive delay (corresponding to 1 year and 6 months). He was subjected to speech therapy due to difficulties related to sensory-motor aspects and oral language. According to a clinical assessment at 4 years of age, despite having communicative intent, language expression was quite restricted. He also had deficits in psychomotor and cognitive executive function that improved with time, especially regarding sensory integration, and he presented an attention deficit. At 5 years of age, an evaluation of language and cognitive development was performed using the Protocol of Observation Behavioral [2]. The examinations revealed the following: limited oral language, with attempts to engage in dialogue; language disorder, especially in expressive language; deficit in motor skills and difficulties in the understanding and execution of tasks and games. With respect to activities of daily living, he showed autonomy in eating and dressing.

**Figure 1:**
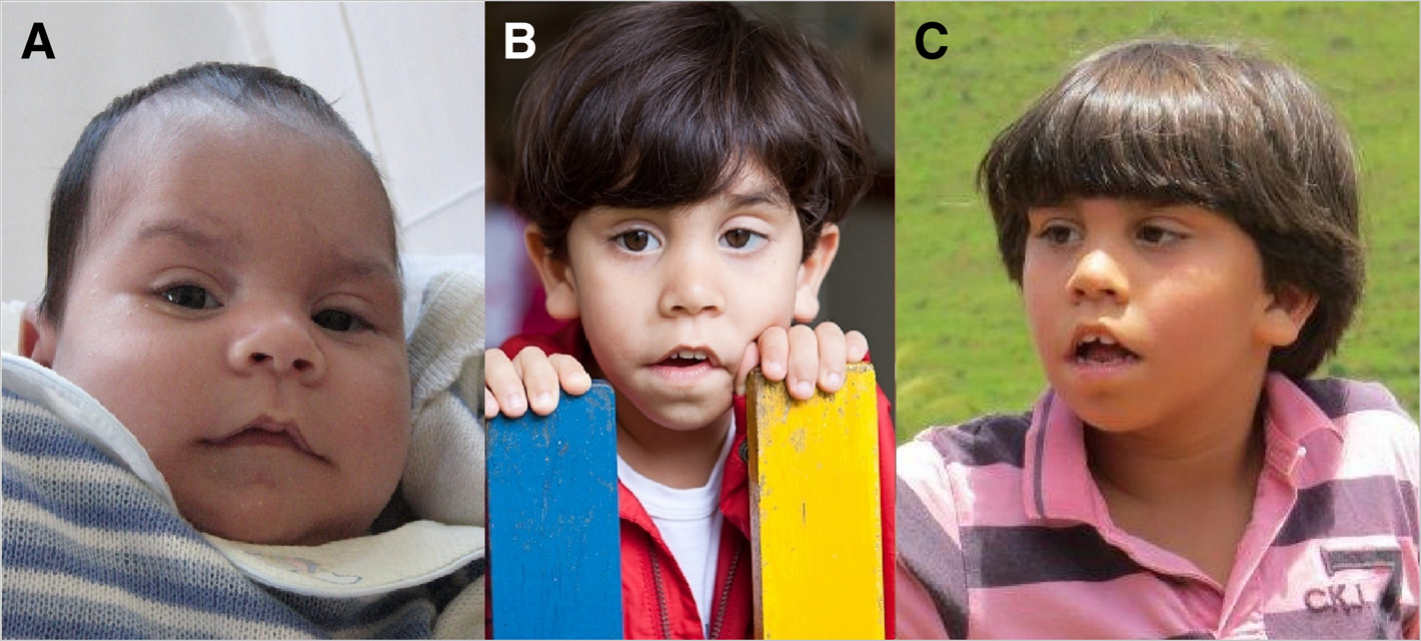
**Patient at the ages of 2 months (A), 3 years and 5 months (B) and 6 years and 10 months (C).** Note the short upturned nose, deep philtrum, thin vermillion of the upper lip and prominent earlobes.

At 6 years and 10 months of age, he measured 125 cm in height (50^th^ – 75^th^ percentile), weighed 26 kg (50-75^th^ percentile) and had an occipital frontal circumference (OFC) of 52 cm (25^th^ – 50^th^ percentile). Dysmorphological examination revealed low-set ears with prominent helices and lobules, hypertelorism (ICD 3.5 cm, >97^th^ percentile), bushy eyebrows, short nose with anteverted nostrils, deep nasolabial furrows, small and open mouth with an open bite and a thin vermilion, a prominent Cupid’s bow of the upper lip and a prominent and everted lower lip. Mild micrognathia was also observed (Figure 1).

The propositus attends a regular school with support. He communicates well with colleagues and teachers, and he is able to express his wishes through language and to understand simple conversation and demands. Further examinations revealed the following: Magnetic resonance imaging (MRI) showed an intact corpus callosum and a small region of ischemia in the glyptic cavity of the cerebellar region. Electroencephalography (EEG) and audiometry results were normal, and he showed no vision problems.

## Results

**Figure 2:**
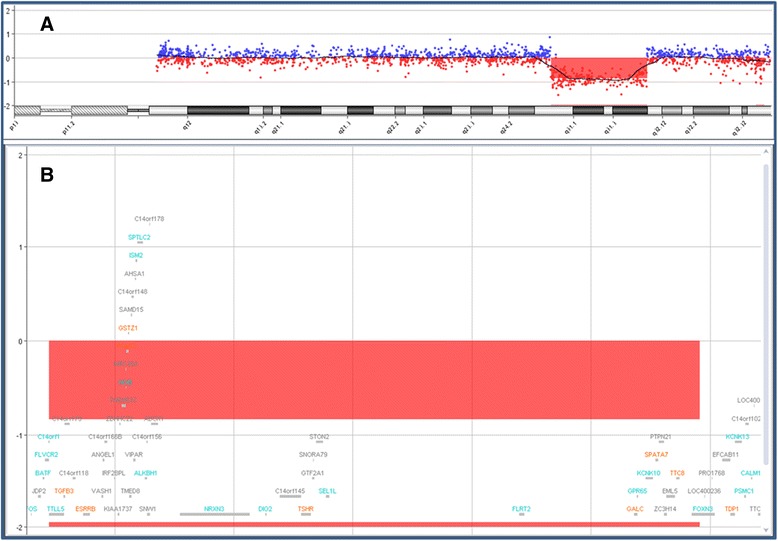
**Chromosome 14 aCGH profile of the patient (in red) and a reference DNA sample from a normal male (in blue).** The figure shows **(A)** a copy number loss corresponding to the segment 14q24.3-q31.3 in a genomic segment with the median log2 ratio shifted to -0.8 (red line). The probe log2 ratios were plotted according to genomic coordinates (based on the UCSC Genome Browser, February 2009, NCBI Build 37 reference sequence). In **(B)**, detail of the 14q24.3-q31.3 region showing the deleted region of ~13 Mb.

With regard to cytogenetic and molecular examinations, the deletion was initially identified through G-banding. The karyotype was interpreted as 46,XY,del(14)(q31). However, fluorescence in situ hybridization (FISH) performed in the proband showed that there was no loss of the terminal region of chromosome 14 (band 14q32). The precise characterization of the deletion using oligonucleotide aCGH showed a loss of genomic material corresponding to an interstitial deletion in the long arm of chromosome 14, segment q24.3-q31.3, of approximately 13.11 Mb (UCSC Genome Browser on Human Feb. 2009 (GRCH37/hg19). Arr 14q24.3q31.3 (76,211,806-89,327,656) ×1 (Figure 2). The parental karyotypes were normal.

## Discussion

Excluding pseudogenes, chromosome 14 presents a mean gene density of 10 genes per Mb (850 genes per 85 Mb), which is close to the mean value estimated for the whole human genome (9.3–10.8 genes per Mb) [3]. Mutation analysis has revealed 21 genes on this chromosome associated with genetic diseases [4]. Among these, Holt-Oram syndrome or a syndrome similar to Holt-Oram syndrome, has been reported in patients with 14q deletions. Furthermore, 70% of those patients fulfilling strict criteria show a mutation in *TBX5* on 12q24.1. Thus, there might be a second locus for this syndrome at 14q23.3q31.1 [5].

The propositus presented with mild to moderate developmental delay, normal growth and a pattern of indistinct minor dysmorphic signs in the absence of organ malformations. For a comparison with previously reported patients with similar or overlapping deletions spanning at least 10 Mb (see Table 1, in order from proximal to distal from the proximal breakpoint), we excluded reports in which the breakpoints were determined only through chromosome banding because it is well known that such methods can provide imprecise determinations leading to incorrect interpretations (for example, a deletion classified as terminal may turn out to be interstitial, as in our proband, and vice versa). Our patient was the first to be subjected to aCGH analysis of the deleted segment. In contrast, using FISH with only a few and mostly flanking markers, it is possible to overlook non-deleted or even duplicated stretches of DNA within a deleted segment, especially in instances of ring chromosomes [6].

**Table 1:** **Comparison of the clinical features of patients with interstitial 14q deletions including the segment 14q23.3 to 14q31.1**

**Feature**	**1 Le Meur**	**2 Zollino, case 25**	**3 Propositus**	**4 Karnitis, Byth**	**5 Cingöz**	**6 Schlade**	**7 Zollino 22**	**Total**
Proximal breakpoint	q23.3	q24.3	q24.3	q24.3	q24.3	q24.3	q24.3	
Distal breakpoint	q31.1	q32.13	q31.3	q31.3	q32.2	q32.13	q31.11	
Size of the deletion in Mb	13.937.056	20.971.834	13.115.851	11.665.854	21.545.793	18.576.858	13.981.234	
Minimal deletion (base pairs)	67.736.534	73.942.355	76.211.806	76.822.337	77.226.431	77.823.431	77.867.749	
	81.673.589	94.914.188	89.327.656	88.488.190	98.771.224	96.400.270	91.848.982	
Technique used	FISH/BACs	FISH/BACs	aCGH	FISH/BACs	FISH/BACs	FISH/BACs	FISH/BACs	
Age at last examination (years)	6/12	18	6 9/12	3 6/12	10	3	19	-
Developmental delay	+	++	+	+	+	+	++	7/7
Intellectual impairment		++	+	+, mild	+	+	++	7/7
Language delay		+	+		+	+	+	6/6
Postnatal growth retardation		+		-	+	+		4/6
Muscular hypotonia	+	+	+		+	+	+	7/7
Microcephaly postnatally		+			-	+	-	¾
Ocular hypertelorism	+		+	+	+			5/5
Inner epicanthic folds			+	+	+	+		5/5
Ptosis of eyelids		+		+	+			3/6
Downslant/palpebral fissures	+		+	+	-			¾
Flat/depressed nasal bridge			+	+	+			3/3
Strabismus		+		+				
Short/bulbous nose	+		+	+	+	+		5/5
Deep/broad philtrum	+		+					2/2
Thin vermilion of the upper lip	+		+	+	+			4/4
High arched palate			+	+	-			2/3
Abnormal dentition			+	+	-			2/3
Micro-/retrognathia	+			+	-	+		¾
Pointed chin			+		+			2/3
Dysplastic/low-set ears	+, asymm.		+	+	+	+		6/6
Scoliosis		+					+	
			+				+	
Congenital heart defect	+	-	-	-	+	-	-	3/6
Hearing impairment	+(?)	+				+		4/4
Findings in single patients	Radial a/hypoplasia cleft palate, mild ventricular dilatation, maternal balanced insertion	Myopia, aggressivity	Cupid’s bow of upper lip	Breech delivery, central lower incisor, transverse palmar crease left	Blepharo-phimosis, facial asymmetry	Brachyda-ctyly, syndactyly 2/3 toes Seizures, Hirsutism	Susceptible to infections	

+: present; -: absent, empty: not described Patients: 1. [5] 2. [7], case 25; 3. Present case; 4. [8,9]; 5. [1]; 6. [10]; 7. [7], case 22.

Table 1 shows that except for the patient described by LeMeur [5], the proximal breakpoints were quite similar in all patients, ranging from bp 73.942.355 [7], patient 25 to 77,867,749 [7], patient 22, whereas the distal breakpoints differed considerably, from 88,488,190 [8,9] to 98.771.224 [1,10]. However, these represent the minimal deleted segments because the space between the last or first present marker and the first or last deleted marker was quite different, and some markers were not precisely mapped. In addition, findings present in only one patient were listed separately, and comparison was limited by the uncertainty of whether dysmorphic findings not described in the different case reports were absent or not considered worth mentioning.

Our comparison of clinical features revealed that neurodevelopmental features such as developmental delay, muscular hypotonia and language delay were quite consistent, as were some frequent and non-specific dysmorphic signs including hypertelorism, inner epicanthic folds, short and bulbous nose with a depressed bridge, thin upper lip and dysplastic ears. However, malformations were inconsistently found, as heart defects were only found in 3/6 patients, and different defects were observed in one patient each. Hearing impairment was reported 4 times, but the other studies did not report on the presence or absence of hearing impairment, and in the reported cases, the type was conductive and sensory-neural, with 2 instances each.

The patient with the greatest accumulation of congenital malformations had at least 6 Mb deleted proximal of the deletion in the other patients [5]. We did not include the following two patients in the comparison. First, Vaags et al. [11] reported a patient with a very small deletion (0.063 Mb) inside that detected in our propositus; this patient had Asperger syndrome with *NRXN3* deletion. Although *NRXN3* is associated with autism [12] and lies inside our reported deletion, our propositus did not display features of autism. Second, Shimada et al. [12] described a 2-year-old boy with growth and severe mental retardation, a dysmorphic pattern comparable to that observed in our patient and MRI findings of leukodystrophy. This deletion spanned 2.0 Mb, of which 0.8 Mb overlapped with the proximal deleted region in our patient. This patient was found to have a deletion of *EIF2B2*, a gene associated with leukodystrophy, which maps outside the deletion in our propositus, who consequently does not show similar neurologic symptoms.

Table 2 lists the genes with only one copy in the propositus. The list shows that mutations of some of these genes, including *TTC8* (Bardet-Biedl syndrome 8), *POMT2* (Walker-Warburg syndrome), *GALC* (Krabbe disease), *GSTS1* (Tyrosinemia Ib), and *SPATA7* (Leber amaurosis 3), produce well-known clinical entities. However, findings characteristic for these conditions are not present in our propositus because hemizygosity would not cause the condition unless the remaining allele carries a mutation or in the case of uniparental disomy. Except for *NRXN3* and *EIF2B2* (see above), the genes listed in Table 2 are not known to cause specific phenotypes if one allele is absent. This is different from more proximal deletions that cause anophthalmia, cataract (*SIX1*, *BMP4*)), hearing loss (*OTX2*), abnormal pituitary function (*SIX4* and *SIX6*) and polydactyly, all due to hemizygosity of the related genes [13-16]. In patients with deletions distal from that in the propositus and mostly terminal, including ring chromosomes, the clinical patterns tend to be indistinct, mostly showing patterns of dysmorphic signs [17,18]. One exception is the case of a mother and daughter who both showed hemifacial microsomia [19]; however, the latter could theoretically be independent of the small deletion.

**Table 2:** **An assembly of genes deleted in the present patient according to the Online Mendelian Inheritance in Man (OMIM) annotation**

**Gene**	**OMIM annotation**	**Name/Possible function**	**Associated syndromes**
FLRT2	604807	Fibronectin-like domain-containing leucine-rich transmembrane protein 2	-
C14orf4	611720	Chromosome 14 open reading frame 4.	-
SPTLC2	605713	Serine palmitoyltransferase, long-chain base subunit 2. Sphingolipid biosynthesis.	Neuropathy, hereditary sensory, type IC.
ESRRB	602167	Estrogen-related receptor beta.	Deafness, autosomal recessive 35.
NRXN3	600567	Neurexin III. Polymorphic cell surface protein expressed in neurons.	-
GPR65	604620	G protein-coupled receptor 65.	-
TGFB3	190230	Transforming growth factor beta-3.	Arrhythmogenic right ventricular dysplasia 1; Rienhoff Syndrome
TTLL5	612268	Tubulin tyrosine ligase-like family, member 5. Role in glucocorticoid-mediated induction and repression.	Cone-rod dystrophy 19
TTC8	608132	Tetratricopeptide repeat domain 8.	Bardet-Biedl syndrome 8.
FOXN3	602628	Forkhead box N3.	-
ISM2	612684	Isthmin 2, zebrafish homolog. Secreted proteins with diverse functions such as cell adhesion, cell angiogenesis, and patterning of developing nervous system.	-
KCNK10	605873	Potassium channel, subfamily K, member 10.	-
POMT2	607439	Protein o-mannosyltransferase 2.	Walker-Warburg syndrome.
DIO2	601413	Deiodinase, iodothyronine, type II. A selenoprotein that catalyzes the 5-prime deiodination of thyroxine (T4) to generate an active thyroid hormone,3,3-prime,5 triiodothyronine (T3).	-
GALC	606890	Lysosomal enzyme involved in the catabolism of galactosylceramide.	Krabbe disease.
GSTZ1	603758	Tyrosinemia, type Ib.	Tyrosinemia, type Ib.
ALKBH1	605345	AlkB, *E. coli* homolog. Protects cells from mutation and cell death caused by SN2-type alkylating agents.	-
NGB	605304	Neuroglobin. Lower resistance to ischemia and neurofibrillary tangles.	Alzheimer disease.
TSHR	603372	Thyroid stimulating hormone receptor	Hyperthyroidism,familial gestational.
SPATA7	609868	Spermatogenesis associated 7	Leber congenital amaurosis 3 Retinitis pigmentosa
SEL1L	602329	Suppressor of lin-12-like (C. elegans)	-

Genomic imprinting could theoretically cause part of the clinical picture of our propositus. Sutton and Schaffer [20] suggested that 14q23-q32 is likely an area where imprinted genes may reside because distinct phenotypes have been associated with both maternal and paternal uniparental disomy (UPD) for this chromosome. However, according to the authors, these features mapped to other segments, and they were not present in the propositus. Unfortunately, we could not determine the parental origin of the deletion in our propositus. Generally, most interstitial deletions are paternal in origin. However, because maternal UPD is correlated with profound intrauterine and postnatal growth deficiency and early puberty, loss of a critical imprinted segment in the region 14q24-q31 is unlikely because the propositus does not show this key feature. In addition, the clinical picture of paternal UPD 14 is more severe and less consistent; therefore, a comparison with our patient was not possible.

## Conclusions

In summary, the clinical findings in our propositus compared to those in patients with similar deletions show that deletion of the segment 14q24.3-q31.3 is accompanied by a pattern of developmental delay, muscular hypotonia and a relatively non-specific pattern of dysmorphic findings. Thus, it would be difficult or even impossible to recognize similar cases on the basis of clinical findings alone.

## Methods

Standard GTG banding was performed at a resolution of 400-550 bands on metaphase chromosomes from peripheral blood lymphocytes of the patient and his parents. Molecular karyotyping was performed on the proband using through whole-genome array-CGH using a 60-mer oligonucleotide-based microarray with a theoretical resolution of 40 kb (8x60K, Agilent Technologies Inc., Santa Clara, CA) (Figure 2A,B). Labeling and hybridization were performed following the protocols provided by the manufacturers. Images of the arrays were taken using a microarray scanner (G2600D) and processed using Feature Extraction software (v9.5.1), both from Agilent. The raw data were analyzed by Agilent Cytogenomics v2.7.8.0 software with the statistical algorithm ADM-2, using a threshold of 6.0 and a 4-probe minimum aberration call.

## Consent

Written informed consent was obtained from the patient’s parents for publication of this Case report and any accompanying images. A copy of the written consent is available for review by the Editor-in-Chief of this journal.
